# Reducing the metabolic energy of walking and running using an unpowered hip exoskeleton

**DOI:** 10.1186/s12984-021-00893-5

**Published:** 2021-06-06

**Authors:** Tiancheng Zhou, Caihua Xiong, Juanjuan Zhang, Di Hu, Wenbin Chen, Xiaolin Huang

**Affiliations:** 1 Institute of Rehabilitation and Medical Robotics, State Key Lab of Digital Manufacturing Equipment and Technology, Huazhong University of Science and Technology, Wuhan, 430074 Hubei China; 2 Institute of Robotics and Automation Information System and the Tianjin Key Laboratory of Intelligent Robotics, Nankai University, Tianjin, 300071 China; 3 Department of Rehabilitation Medicine, Tongji Hospital, Tongji Medical College of Huazhong University of Science and Technology, Wuhan, 430074 Hubei China

**Keywords:** Metabolic reduction, Human walking and running, Hip unpowered exoskeleton, Hip flexion, Human response

## Abstract

**Background:**

Walking and running are the most common means of locomotion in human daily life. People have made advances in developing separate exoskeletons to reduce the metabolic rate of walking or running. However, the combined requirements of overcoming the fundamental biomechanical differences between the two gaits and minimizing the metabolic penalty of the exoskeleton mass make it challenging to develop an exoskeleton that can reduce the metabolic energy during both gaits. Here we show that the metabolic energy of both walking and running can be reduced by regulating the metabolic energy of hip flexion during the common energy consumption period of the two gaits using an unpowered hip exoskeleton.

**Methods:**

We analyzed the metabolic rates, muscle activities and spatiotemporal parameters of 9 healthy subjects (mean ± s.t.d; 24.9 ± 3.7 years, 66.9 ± 8.7 kg, 1.76 ± 0.05 m) walking on a treadmill at a speed of 1.5 m s^−1^ and running at a speed of 2.5 m s^−1^ with different spring stiffnesses. After obtaining the optimal spring stiffness, we recruited the participants to walk and run with the assistance from a spring with optimal stiffness at different speeds to demonstrate the generality of the proposed approach.

**Results:**

We found that the common optimal exoskeleton spring stiffness for walking and running was 83 Nm Rad^−1^, corresponding to 7.2% ± 1.2% (mean ± s.e.m, paired t-test p < 0.01) and 6.8% ± 1.0% (p < 0.01) metabolic reductions compared to walking and running without exoskeleton. The metabolic energy within the tested speed range can be reduced with the assistance except for low-speed walking (1.0 m s^−1^). Participants showed different changes in muscle activities with the assistance of the proposed exoskeleton.

**Conclusions:**

This paper first demonstrates that the metabolic cost of walking and running can be reduced using an unpowered hip exoskeleton to regulate the metabolic energy of hip flexion. The design method based on analyzing the common energy consumption characteristics between gaits may inspire future exoskeletons that assist multiple gaits. The results of different changes in muscle activities provide new insight into human response to the same assistive principle for different gaits (walking and running).

**Supplementary Information:**

The online version contains supplementary material available at 10.1186/s12984-021-00893-5.

## Background

Walking and running are the most common means of locomotion in human daily life. Through evolution over generations, the human anatomical bases have been well shaped to both walking and endurance running [1]. However, the efficiencies of positive work (positive mechanical power/net metabolic power) during walking and low-speed running are still not perfect, only 0.26–0.35 and 0.35–0.41 respectively [2], which is one of the direct factors that affect human locomotion performance. Therefore, people have been searching for different ways to enhance energy efficiency and thus reduce the metabolic energy of walking and running.

Over the past six years, great progress has been made in the study of exoskeletons for reducing the metabolic rate of walking or running [3]. In studies on exoskeletons for walking assistance, both autonomous powered [4–6] and unpowered [7–9] exoskeletons were demonstrated to reduce metabolic cost. It has been found that the assistance of the net mechanical power input of the powered exoskeletons at specific phases, such as during ankle push-off [4, 5] or hip flexion/extension [6] in the stance phase, can decrease the biological positive joint power, and thus reduce the overall metabolic cost during walking. As alternatives, unpowered exoskeletons, which exploit springs to assist humans in recycling energy [7, 8] and transferring energy [9] more efficiently, reduced the metabolic rate by improving the human-exoskeleton energy efficiency as a whole. There have also been breakthroughs in unpowered [10, 11], tethered powered [12] and autonomous powered exoskeletons [13, 14] that reduce the metabolic rate of running. Unpowered exoskeletons [10, 11], which were designed based on a biomechanical analysis of running, enhance the energy efficiency during specific gait phases while simplifying the exoskeleton structure to reduce the metabolic penalty caused by mass. Lee et al. found that the metabolic cost of running can be reduced with a simulation-optimized actuation profile using a tethered soft exosuit [12]. Kim et al. proposed an online detection algorithm that enables the soft exosuit to switch seamlessly between walking and running [13]. These two studies provide a solid foundation for the soft exosuit to reduce the metabolic cost of both walking and running by 9.3% and 4.0% respectively [14]. This is also the only autonomous powered exoskeleton that can reduce the metabolic rates of both walking and running. To date, the studies on exoskeletons that can reduce the metabolic cost of both walking and running are limited.

Several critical factors may be obstacles to enhancing the economy for both walking and running. One of the challenges is how to overcome the fundamental biomechanical differences between walking and running [14]. With the increasing speed, humans spontaneously transit from walking like an inverted pendulum [15] to the more bouncing gait of running [16], and both muscle behavior [17, 18] and joint mechanical power [2] show significant changes, which may result in different assistive approaches or different optimal assistance magnitudes and timings [10, 19]. However, unlike the hip exosuit [14], which can provide customized assistance based on differences in the natural COM fluctuation of participants, most exoskeletons have been designed based on the biomechanical analysis of walking or running, resulting in most exoskeletons providing effective assistance only for specific gaits. The unpowered ankle exoskeleton [7], which uses a spring-clutch mechanism to reduce the muscle effort of plantar flexors during the mid-stance phase, was demonstrated to reduce the metabolic rate of walking by 7.2%. On the contrary, the authors found that the energy expenditure was increased by 11.1% when this passive assistive principle was applied to running scenarios [19]. The primary reason might be the different energy consumption characteristics of ankle joint between slow walking and moderate-speed running [20]. The benefits of passive assistance may be limited by the fact that the ankle performs significantly more positive mechanical work than negative mechanical work in the late stance phase during running. Similarly, the hip unpowered exoskeleton, which exploits a torsional spring to recycle contralateral hip joint energy to assist both hip flexion and hip extension, can reduce the metabolic cost of running by 8% but was found to be ineffective in walking [10]. As the hip joint does not produce positive power during the late swing phase of walking, which is different from running, the hip extension assistance of the exoskeleton interfered with the natural biomechanics of hip muscle during passive leg swing in process of walking.

Another challenge is how to minimize the metabolic penalty of the added exoskeleton mass while providing effective assistance for both gaits. Previous work showed that every kilogram of mass added to the lower limb segments from proximal to distal to trunk results in 1.4–4.4 times the metabolic penalty during running than the during walking (summarized in the Additional material of [14]). In the previous studies on exoskeletons, researchers reduced the metabolic penalty by using lightweight soft materials to construct the exoskeleton frame [21], concentrating the mass of motor combinations and the battery close to the trunk and transferring assistive power to the distal joint through a remote transmission mechanism [21, 22]. However, the metabolic penalty of the exoskeleton mass was still not negligible for the powered exoskeleton. Although the best-in-class hip exo-suit [14] was demonstrated to benefit both walking and running, the metabolic reduction for running was significantly lower than the for walking. One of the most likely reasons might be that the assistance effect was partly offset by the greater metabolic penalty of the same exoskeleton mass.

**Fig. 1:**
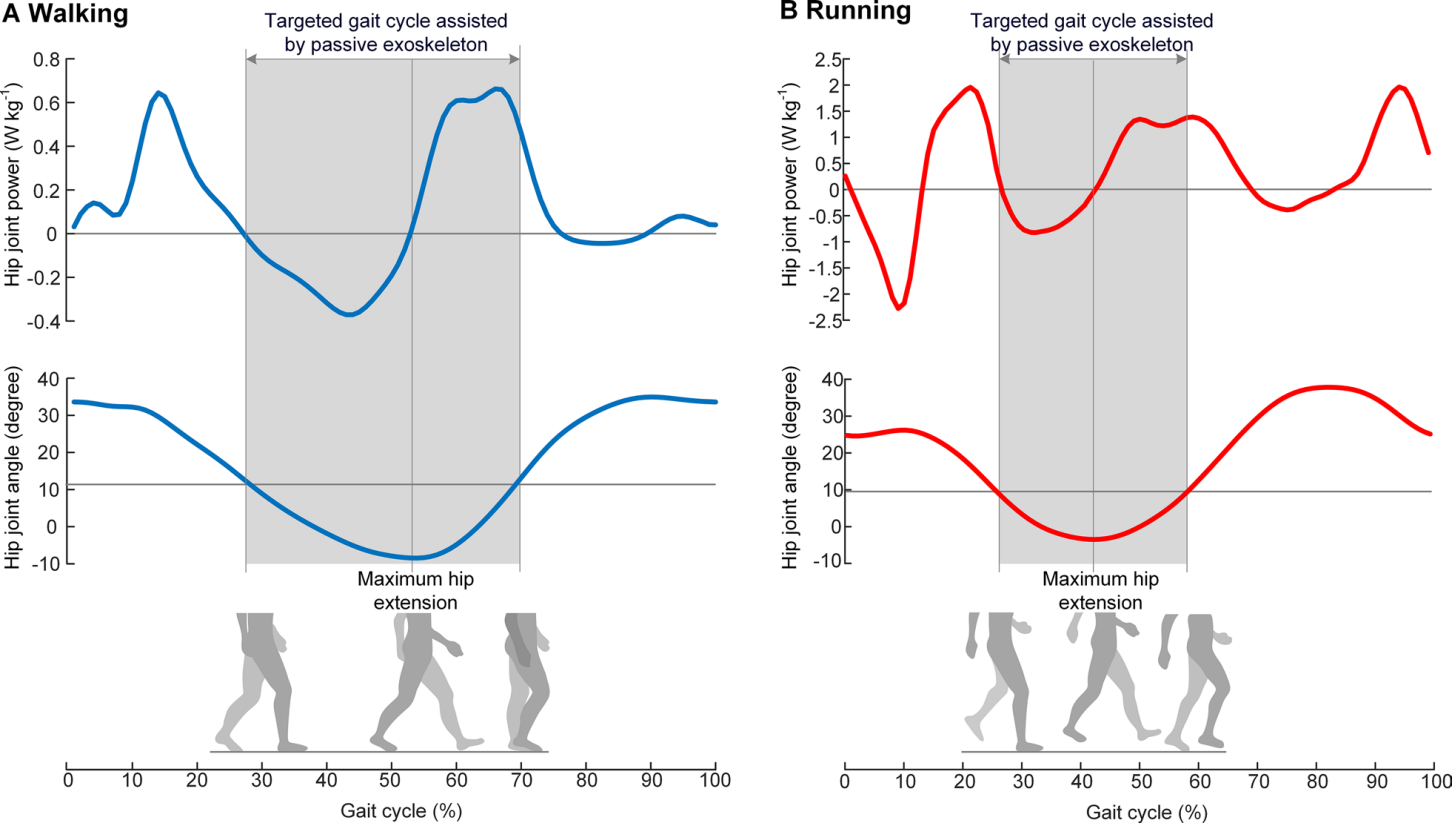
Hip joint power during walking and running. In the shaded interval of both walking and running, the hip joint extends from about 10 degrees of flexion position to the maximum extension position, corresponding to a period of negative power; Then, the hip joint flexes from the maximum extension position, corresponding to a period of positive work. The dataset of walking is the mean value of 7 participants (mean ± s.t.d; age, 26.9 ± 3.0 years; height,169.2 ± 7.0 cm; weight, 66.3 ± 14.2 kg), which is reported by [42]; The dataset of running is the mean value of 7 participants (mean ± s.t.d; age, 32.7 ± 5.6 years; height, 176.9 ± 6.2 cm; weight, 67.0 ± 8.9 kg), which is reported by [43]

**Fig. 2:**
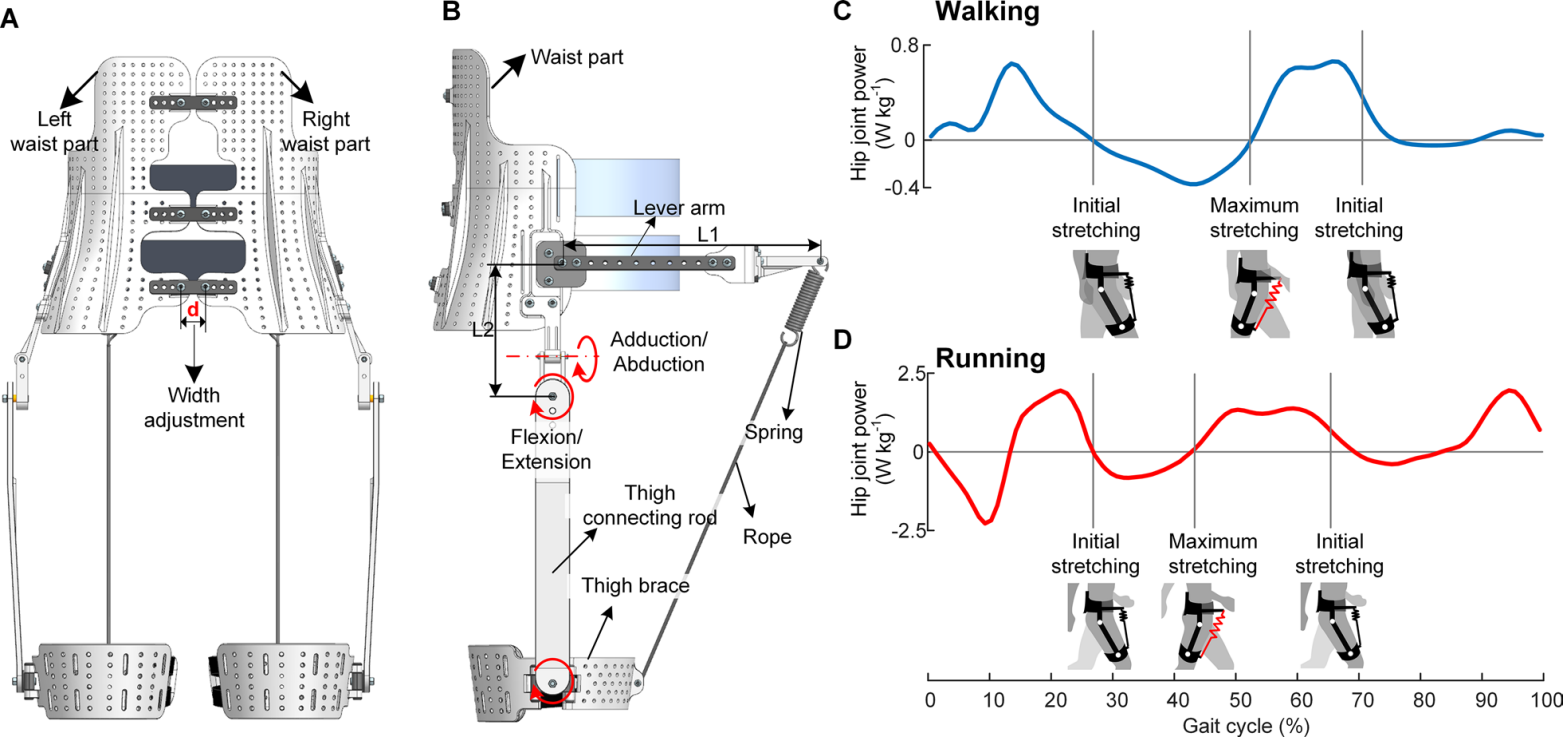
Exoskeleton components and working process of the exoskeleton. **A** Back view of the exoskeleton. We change the length of d to adjust the width of the two waist parts for best-fit participants. **B** Right view of the exoskeleton. The waist part and thigh connecting rods were connected using two rotary joints with plain bearing in series, allowing the adduction/abduction and flexion/extension of the hip joint. **C** and **D** Working process of the exoskeleton. The assistance interval is in accordance with the hip joint negative and positive mechanical power during walking and running. In the walking and running condition, the assistance started at nearly 10 degrees of hip flexion position. During the negative power period of the hip joint, the spring stores energy with the hip extension to its maximum extension position. During the positive power period of the hip joint, the spring releases the stored energy to assist hip flexion to 10 degrees of hip flexion position

As mentioned above, the combined requirements of overcoming fundamental biomechanical differences between the two gaits and minimizing the metabolic penalty of the exoskeleton mass make it challenging to develop an exoskeleton that can reduce the metabolic energy for both gaits. In this paper, we first analyze the common energy consumption characteristics of walking and running (Fig. 1) to find a universal assistive principle for both gaits. Then we propose a hip unpowered exoskeleton (Fig. 2A and B) to regulate metabolic energy of hip flexion during the common energy consumption period of both walking and running. The proposed exoskeleton uses exo-tendons acting in front of the hip joint to recycle the negative mechanical energy and release stored energy to assist hip flexor, which are in accordance with the biological negative mechanical power interval and positive mechanical power interval respectively (Fig. 2C). The lightweight structure, close to the human trunk, minimizes the metabolic penalty caused by the exoskeleton mass. To determine the best assistance magnitude and evaluate human response to the proposed assistive principle, the metabolic rates, muscle activities and spatiotemporal parameters of nine healthy participants are measured and statistically analyzed. We also perform speed experiments to test the influence of locomotion speed on the assistance effect. To demonstrate the generality of the proposed exoskeleton, we measure and compared the metabolic rate of walking/running with the common optimal spring stiffness to that of walking/running without the exoskeleton.

## Methods

### Analysis of common energy consumption characteristics between walking and running

During walking and running, both the ankle and hip musculature serve as the major contributors to the production of positive mechanical work [2]. However, the hip musculature consumes more metabolic energy to produce each joule of positive work than does the ankle musculature, as the energy efficiency of the hip musculature is significantly lower than that of the ankle musculature [23]. Therefore, improving the energy efficiency of the hip musculature may be essential to reduce the metabolic energy of walking and running. During walking and running, the intrinsic power curves of the hip joint show a similar pattern during the late stance phase and early swing phase with a negative mechanical power interval followed by a positive mechanical power interval (Fig. 1). Previous studies indicated that part of the energy required for performing positive mechanical work comes from the energy stored by stretching tendons in the previous period of negative work, thus forming an intrinsic energy-saving mechanism [24]. However, learning from the kinesiology and anatomy of the lower limb [25], the ankle plantar-flexors insert through the collagen-rich Achilles tendon (as shown in Additional file 1: Fig. S1), which acts like a spring to recycle energy and compensate muscle work efficiently [26]. In contrast, most hip flexors do not have such long series elastic components. When the hip flexors perform negative mechanical work, the peak energy efficiency is − 120%, as the friction of muscle bundles also causes energy dissipation [26]. Therefore, the energy-saving mechanism of hip musculature may not be as perfect as that of ankle musculature [24]. Suppose we provide the hip joint with an external loop that regulates metabolic energy of hip joint by efficiently recycling part of the negative mechanical energy and releases the stored energy to partly replace muscle effort to produce positive work. In that case, it is likely to reduce both the energy dissipation and positive mechanical work produced by the hip flexors. In our previous simulation study, we added an exoskeleton spring (exo-tendon) to assist hip flexors in recycling energy. The simulation results indicated that the biological efforts of hip flexors and metabolic cost were reduced with the assistance of exo-tendon during walking [8]. As analyzed above, the similarity of hip biomechanics and kinematics between the two gaits makes it possible to develop an exoskeleton with a universal assistive principle for both walking and running.

### Design of an unpowered hip exoskeleton

In this study, we designed an unpowered hip exoskeleton that passively provides hip flexion assistance during the common energy consumption period of both walking and running. The proposed exoskeleton uses exo-tendons acting in front of the hip joints to recycle the negative mechanical energy and release stored energy to assist hip flexor, which is in accordance with biological negative mechanical power interval and positive mechanical power interval respectively (Fig. 2). The hip unpowered exoskeleton consisted of a waist frame, a waist belt, force leverage, thigh connecting rods and thigh braces (Fig. 2A and B). We used modified orthotics methods and 3D printing methods to fabricate the waist and thigh braces of the exoskeleton for the best fit for the human irregular surface. The width between the left waist part and right waist part can be adjusted to fit individuals. The waist frame and thigh connecting rods were connected using two rotary joints with plain bearing in series, allowing the adduction/abduction and flexion/extension of the hip joint. A rotary joint connects the thigh connecting rod and thigh brace with consideration for wearing comfort.

According to anatomical knowledge, the primary hip flexors include the iliopsoas, sartorius and rectus femoris. The iliopsoas, which consists of the iliacus and psoas major, is a potent hip flexor not only for femoral-on-pelvic hip flexion but also for pelvic-on-femoral hip flexion [25]. The proximal attachments of the psoas major are along the transverse processes of the last thoracic and lumbar vertebrae. The distal end of the iliopsoas is attached to the femur through the broad tendon. The deflection of the tendon raises the angle-of-insertion, thus increasing the muscle leverage for hip flexion [25]. Inspired by this biological anatomical structure, we also use a lever arm (Fig. 2B) on the proximal end of the spring to amplify the torque produced by the spring. The lever is 0.14 m (L2) above the exoskeleton hip joint, a similar height to the proximal end of the iliopsoas. The length of the lever arm was set to 0.27 m (L1) with the combined consideration of wearability for overweight participants and decreasing the metabolic penalty of lever arm mass. As shown in Fig. 2C, we adjust the length of rope to make the initial stretching of spring at an approximately 10-degree hip flexion position. The exoskeleton spring recycles part of the negative mechanical energy with the hip extension and then releases the stored energy to assist hip flexion.

To determine the optimal assistance magnitude, we chose four sets of springs with the stiffnesses of 1.1, 2, 3, 3.8 k N m^−1^ based on the results of our preliminary experimental results [8]. Spring stiffnesses were obtained by the KATALOG company with testing experiments. As the average radius of the springs is 0.19 m, the average exoskeleton rotational stiffnesses are 40, 72, 108, 137 Nm rad^−1^, within the range of hip joint quasi-stiffness during the late stance phase of walking [27]. The detailed component mass of the exoskeleton is presented in Table 1.

**Table 1:** Unpowered hip exoskeleton mass distribution

Segment	Mass (g)
Waist frame	760
Lever arm	235
Average spring (× 2)	68
Hip joints (× 2)	135
Thigh segments (× 2)	107
Total mass (biarticular sum)	1305

### Participants

Nine healthy male adults with no history of musculoskeletal diseases and no gait abnormalities (age 24.9 ± 3.7 years, weight 66.9 ± 8.7 kg, height 1.76 ± 0.05 m, mean ± standard deviation) participated in the study. The study was approved by the Chinese Ethics Committee of Registering Clinical Trials. All participants signed written informed consent before the experiment. The participants whose images appear in the manuscript have provided written consent for the publication of their images according to the policies of the Journal of NeuroEngineering and Rehabilitation.

### Experimental protocol

**Fig. 3:**
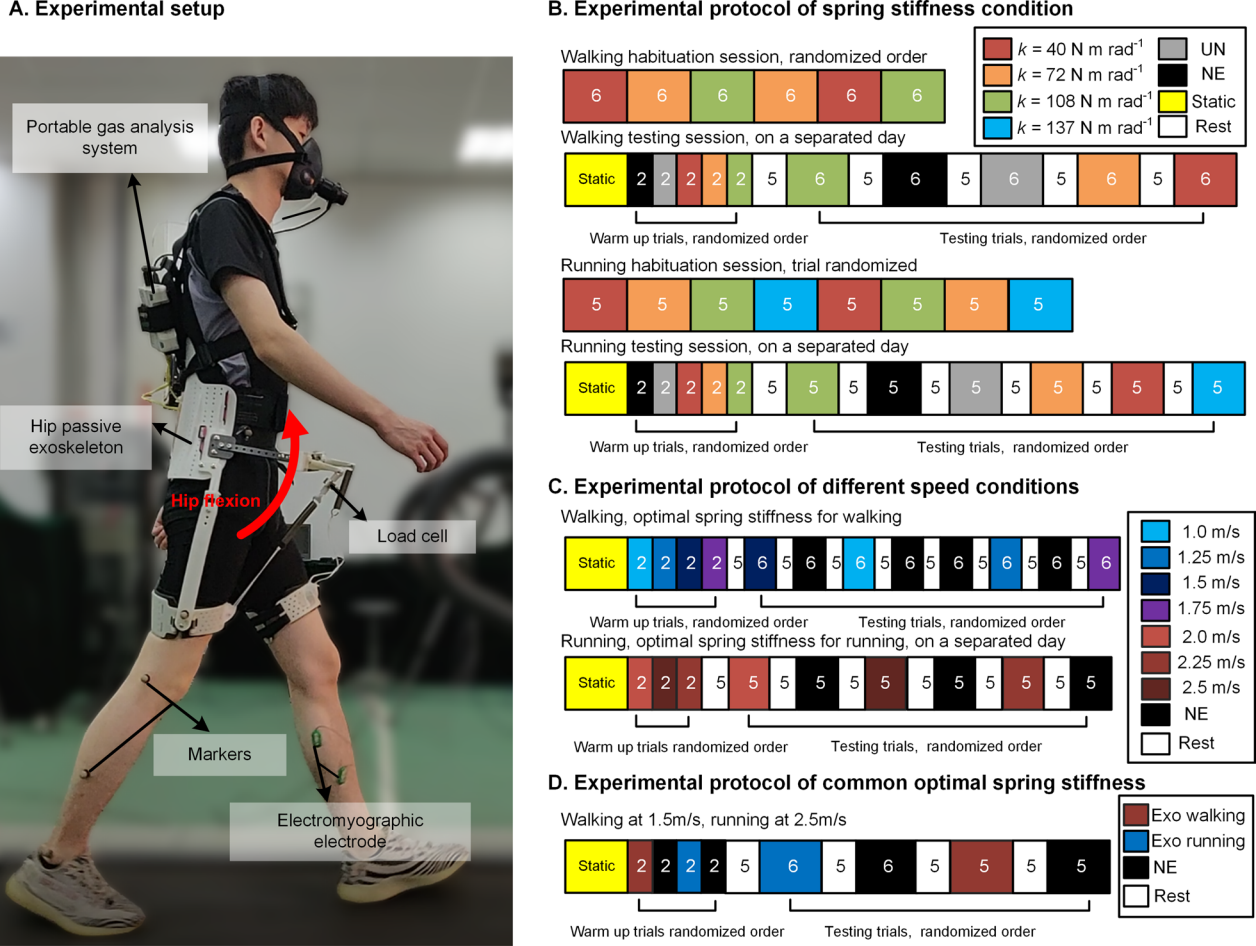
Experimental setup and metabolic reductions. **A** Experimental setup. While a participant was walking/running with the hip unpowered exoskeleton, the spring acted in front of the hip joint to provide hip flexion torque passively. The passive force of spring (load cell), lower limb segment motion (Vicon motion capture system), muscle activities (electromyography system) and metabolic rate (indirect calorimetry) were measured. **B**–**D** Experimental protocol of the three experiments. Each experiment was performed on a separated day to prevent fatigue. The number on the figure indicates time duration of each trial. The NE condition of **C** refers to walking/running without exoskeleton at different speeds

The testing protocol includes three main experiments (Fig. 3). In the first experiment, we tested the metabolic and biomechanical effect of exoskeleton on nine male subjects walking and running on a treadmill at a constant speed of 1.5 m s^−1^ and 2.5 m s^−1^ respectively. Participants walked with the selected spring stiffnesses to determine the optimal spring stiffness for metabolic reduction. After obtaining the optimal spring stiffnesses (72 Nm Rad^−1^ for walking and 108 Nm Rad^−1^ for running), we recruited the same 9 participants to walk/run with the optimal spring stiffness at the speed of 1.0, 1.25, 1.5, 1.75, 2.0, 2.25, 2.5 m s^−1^. In the second experiment, the aim was to find the relationship between metabolic reduction and walking/running speeds. In the third experiment, the objective is to demonstrate that the metabolic rates of both walking and running can be reduced with the common optimal stiffness spring found in the first experiment. The detailed experimental protocols are stated as follows.

In the first experiment (Fig. 3B), the walking and running experiments involved walking habituation session, walking testing session, running habituation session and running testing session. Each session was performed on a separated day to avoid fatigue effects. In the habituation sessions, participants adapted themselves to the exoskeleton with different spring stiffness. They experienced each exoskeleton spring stiffness twice. Each walking trial lasted 6 min and each running trial lasted 5 min. Participants rested as long as they need between each habituation trial. In the testing sessions, participants first performed a 6-min standing trial for basic metabolic rate data, then warm-up trials and testing trials. In the walking testing session, the warm-up trials involved five conditions: 3 spring stiffnesses conditions (40, 72, 108 Nm Rad^−1^), walking with no exoskeleton condition (NE) and walking with no assistance condition (UN). Each condition lasted 2 min. After a 5-min break, participants performed five 6-min testing conditions: 40, 72, 108 Nm Rad^−1^, NE and UN for biomechanical and metabolic data. The participant took a 5-min rest between each testing trial. We analyzed experimental data in the last 2 min of each trial. In the running testing session, the warm-up trials involved six conditions: 4 spring stiffnesses conditions (40, 72, 108, 137 Nm Rad^−1^), a running with no exoskeleton condition (NE) and a running with no assistance condition (UN). Each spring stiffness condition lasted 2 min. After a 5-min break, participants performed six 5-min testing conditions: 40, 72, 108, 137 Nm Rad^−1^, NE and UN for biomechanical and metabolic data. The participant took a 5-min rest between each testing trial. We analyzed experimental data in the last 2 min of each trial. In both walking and running testing sessions, the order of experimental conditions was randomized to minimize the effect of the order.

In the second experiment (Fig. 3C), walking trails and running trials were performed on separated days to prevent the fatigue effect. Participants first performed a 6-min standing trial for basic metabolic rate data and warm-up trials. The warm-up trials involved walking/running with exoskeleton at selected speeds. Each speed lasted 2 min. After a 5-min rest, participants performed different speed testing conditions for metabolic data. Each walking speed condition lasted 6 min and each running speed condition lasted 5 min. The participant took a 5-min rest between each testing trial. The order of walking/running speed was randomized to minimize the effect of the order. We analyzed experiment data in the last 2 min of each trial.

After the first experiment, we used the intersection of the polynomial functions of walking and running to find optimal exoskeleton spring stiffness for reducing metabolic rates of both walking and running (see Data collection). The theoretical common optimal spring stiffness is 2.3 kN m^−1^ (83 Nm Rad^−1^). In the third experiment (Fig. 3D), participants (N = 9) first performed a 6-min standing trial for basic metabolic rate data and warm-up trials. The warm-up trials involved walking/running with 2.3 kN m^−1^ spring, walking/running without exoskeleton. Each condition lasted 2 min. After a 5-min rest, participants performed four conditions for metabolic data: walking with 83 Nm Rad^−1^, walking without exoskeleton, running with 83 Nm Rad^−1^, running without exoskeleton. Each walking condition lasted 6 min and each running condition lasted 5 min. The participant took a 5-min rest between each testing trial. The order of walking/running speed was randomized to minimize the effect of the order. We analyzed metabolic data in the last 2 min of each trial.

### Data collection

We used an indirect calorimetry system (Oxycon Mobile, CareFusion) to measure oxygen consumption and carbon dioxide production during walking/running. Lower limb motions were measured by a reflective marker motion capture system (Vicon, Oxford Metrics;100 Hz). The markers are attached to human according to [28]. Lower limb muscle activities (soleus, medial gastrocnemius, anterior tibialis, rectus femoris, semitendinosus) were recorded by an electromyography system (SX230, Biometrics, Newport, UK). Electromyographic electrodes are attached to the human body according to [29]. The force produced by the spring was measured by a single-axis load cell (Forsentek FL25).

Lower limb joint angles were calculated from measured lower limb motions using inverse kinematics by software (PlugInGait algorithm, Nexus, Vicon). Muscle activity was band-pass filtered (20–460 Hz) in the electromyography system, rectified and low-pass filtered (fourth-order Butterworth, cut-off frequency 6 Hz) in the software (MATLAB 2018b, Mathworks). Metabolic rate was calculated from the average of carbon dioxide and oxygen rate in the last 2 min using a Brockway equation [30]. The metabolic rate was first normalized by participant weight. The net metabolic rate was obtained by subtracting the metabolic rate of quiet standing from the total metabolic rate of walking/running.

Exoskeleton torque, joint angle and muscle activity of each condition were divided into gait cycles, defined as the period between the heel strike to the next heel strike of the same leg. For each participant, average muscle activity was calculated as the time integral of muscle activity divided by gait cycles in each condition. We normalized the muscle activity of each muscle by dividing the maximum value during the NE condition. After obtaining the average muscle activity, exoskeleton torque and joint angles for each participant, the data were averaged across participants for each condition. The exoskeleton torque was obtained by multiplying the spring force by the calculated lever length (D_lever_). The detailed calculation process of exoskeleton torque is presented in the Additional file 1: Calculation process of exoskeleton torque. We multiply the exoskeleton torque by hip angular velocity to obtain the exoskeleton power. Then we integrated the positive and negative portion of exoskeleton power and divided gait cycle time to obtain average positive and negative exoskeleton power.

### Statistics

In the first experiment, we first performed a Jarque–Bera test (α = 0.05; Matlab 2018b) on the net metabolic data, average muscle activity, average exoskeleton torque, average exoskeleton torque and peak joint angles. The results (*p* > 0.05) showed that these data followed the normal distribution respectively. Then, mixed-model, two-way ANOVA (random effect: participant; fixed effects: spring stiffness) was conducted on net metabolic reduction, average muscle activity, average exoskeleton torque, average exoskeleton torque and peak joint angles across conditions (Walking: 3 stiffness conditions, UN and NE condition; Running: 4 stiffness conditions, UN and NE condition) to verify the effect of spring stiffness (significance level α = 0.05; Matlab 2018b, Mathworks). For each condition, means and standard errors of net metabolic reduction and average muscle activity were calculated across participants. After the ANOVA test, we used a two-sided paired t-test with Holm-Šidák correction to compare spring conditions to no exoskeleton (NE) condition to find which spring condition had a significant change in metabolic rate, average muscle activity, average exoskeleton torque, average exoskeleton torque and peak joint angles. For the net metabolic reduction of walking and running, we also used two least-squares regressions to fit third-order polynomial functions relating mean metabolic reduction to spring stiffness. The F-test was performed on the regression model to determine whether the mean metabolic reduction and spring stiffness had a significant relationship. The intersection of the polynomial functions of walking and running was used to indicated common optimal spring stiffness for metabolic reduction of both walking and running.

In the second experiment, another Mixed-model, two-way ANOVA (random effect: participant; fixed effects: walking/running speed) was conducted on net metabolic rate across speed conditions to test the effect of assistance during different speeds. We used a least-squares regression to fit a third-order polynomial function relating metabolic reduction to walking/running speeds. Then we performed *F*-test on the regression model to determine whether there is a significant cubic relationship between metabolic reduction and walking/running speeds.

In the third experiment, we used a two-sided paired t-test to compare 83 Nm Rad^−1^ condition to no exoskeleton (NE) condition to find which spring condition had a significant reduction in metabolic rate.

## Results

### Metabolic rates

**Fig. 4:**
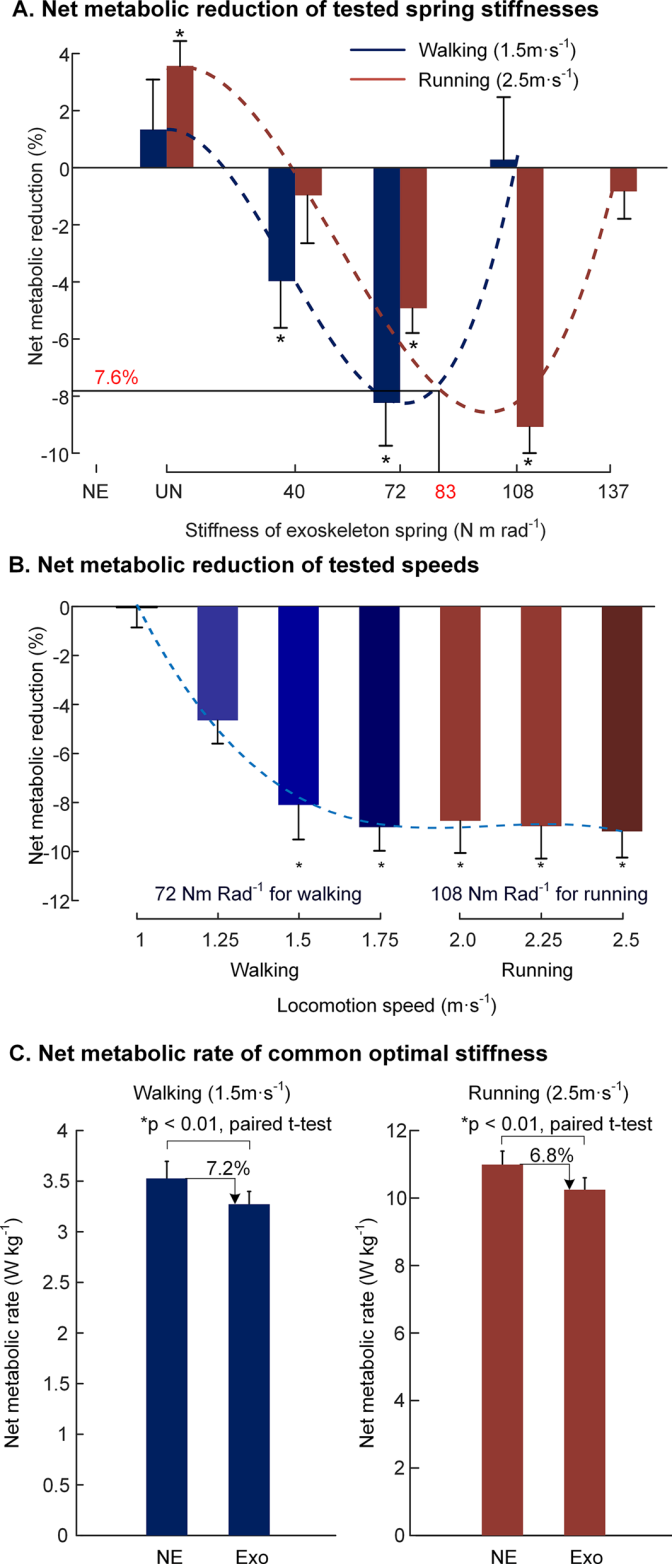
Changes in net metabolic rate. **A** Net metabolic reduction in stiffness experiment. The metabolic reduction of NE condition (control group) was set as zero level. The blue color indicates walking conditions, and red indicates running conditions. The blue and red dashed lines are the third-order best fit to mean metabolic reduction from stiffness conditions in walking and running (*p = 0.007; *p = 0.037). **B** Net metabolic reduction in speed experiment. The metabolic reduction of NE condition (control group) was set to zero level. The figure shows the metabolic reduction during walking and running at different speeds. The blue dashed line is the third-order best fit to mean metabolic reduction from speed conditions in walking and running (*R*^2^ = 0.99, *p < 0.001). **C** Net metabolic changes in common optimal stiffness experiment. The net metabolic rates of walking and running with the 83 Nm Rad^−1^ were reduced by 7.2% ± 1.2% (mean ± s.e.m, paired t-test, p < 0.01) and 6.8% ± 1.0% (p < 0.01) respectively compared to walking/running NE condition. The asterisk symbol indicates a statistically significant reduction compared to NE condition

In the first experiment, as shown in Fig. 4A, we found that the optimal exoskeleton spring stiffnesses for walking and running were 72 N m Rad^−1^ and 108 N m Rad^−1^ respectively in terms of metabolic reduction. With the assistance of the optimal stiffness spring, the metabolic rates of walking and running were reduced by 8.2% ± 1.5% (mean ± s.e.m, two-sided paired t-test with correction for multiple comparisons: *p* < 0.01) and 9.1% ± 1.3% (*p* < 0.01) respectively. The blue dashed line is a cubic best fit to mean net metabolic reduction from spring stiffness (N = 9; y = 4.865e^−7^x^3^ – 5.350e^−5^x^2^ + 0.014; y (W kg^−1^), x (Nm Rad^−1^); *R*^2^ = 0.9998; F-test on the regression model, *p* = 0.015). The red dashed line is a cubic best fit to the mean net metabolic reduction from the spring stiffness (N = 9; y = 2.552e^−7^x^3^ – 3.760e^−5^x^2^ + 0.038; y (W kg^−1^), x (Nm Rad^−1^); *R*^2^ = 0.9647; *p* = 0.035). By obtaining the intersection of the optimal fitting curve, we found that the common optimal stiffness was 83 N m Rad^−1^ and the corresponding metabolic reduction for both walking and running was theoretically 7.6%.

In the second experiment, as shown in Fig. 4B, the metabolic reduction increased with the increasing walking speed (two-way mixed model ANOVA, *p* < 0.001). With the increasing running speed within the tested range, the metabolic reduction did not show significant changes (*p* = 0.97). The relationship between the walking/running speeds and net metabolic reduction during walking/running can be best fit to a cubic polynomial model (N = 9; y = − 0.0741x^3^ + 0.4663x^2^ − 0.9709x + 0.5795; y (W kg^−1^), x (N m Rad^−1^); *R*^2^ = 0.99; *F*-test on the regression model, *p* < 0.001).

As shown in Fig. 4C, the metabolic rates of walking and running with 83 Nm Rad^−1^ were reduced by 7.2% ± 1.2% (mean ± s.e.m, paired t-test p < 0.01) and 6.9% ± 0.8% (p < 0.01) compared to those of walking and running without the exoskeleton. This result demonstrated the generality of the proposed exoskeleton for benefiting both walking and running. The detailed net metabolic rates are presented in the Additional file 1: Table S1, Tables S2 and S3.

### Exoskeleton torque and power

**Fig. 5:**
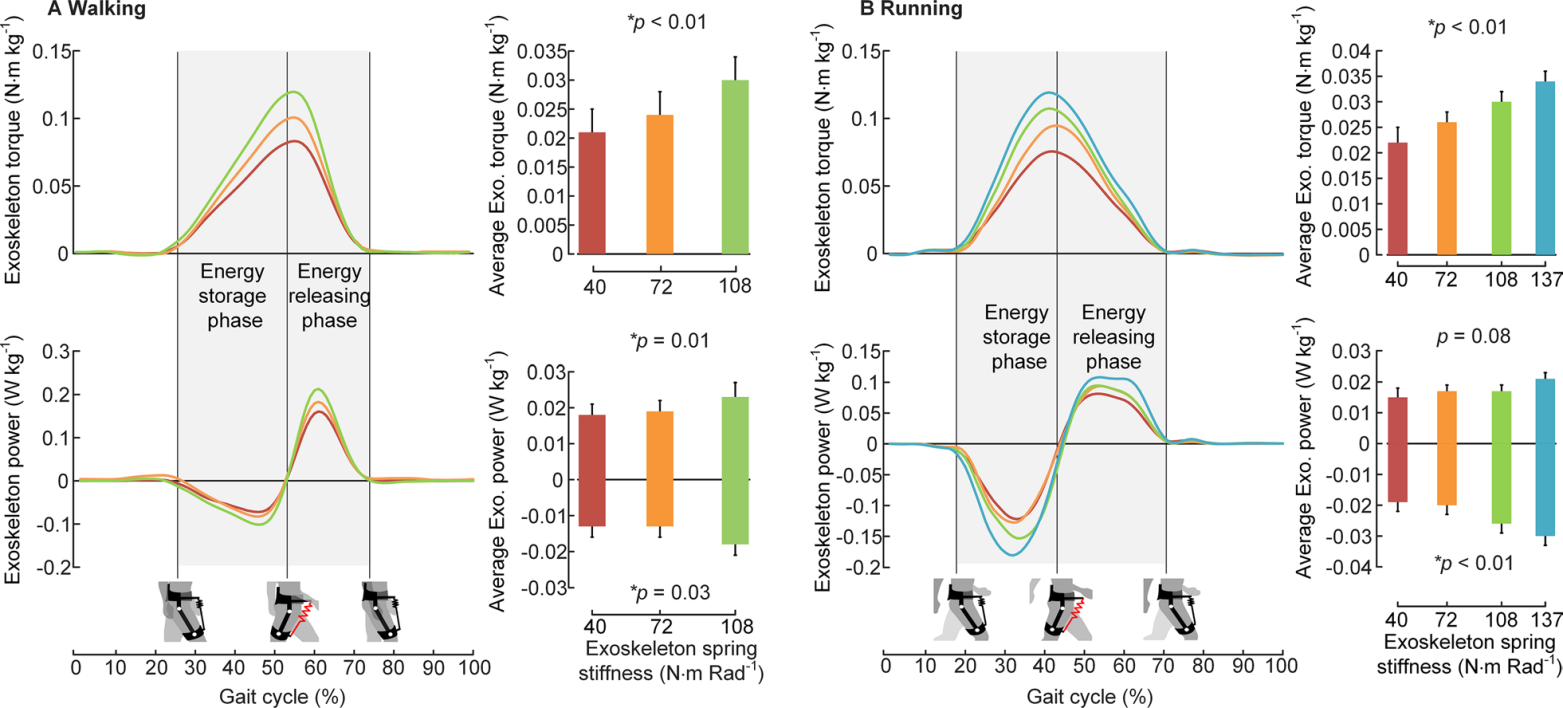
Exoskeleton torque and exoskeleton power. **A** and **B** The exoskeleton torque curves and power curves, which are normalized to body weight for each spring stiffness condition, averaged across participants during walking and running. The bar graphs on the right of the curves indicate the average exoskeleton torque, average exoskeleton positive and negative power. *N* = 9; error bars, s.e.m; p values are the results of mixed model two-way ANOVA (random effect: participant; fixed effect: spring stiffness)

As shown in Fig. 5, the average exoskeleton torque increased with the increasing spring stiffness during walking and running (mixed model two-way ANOVA, *p* < 0.001, *p* < 0.001). Both the average positive power and average negative power of the exoskeleton increased with increasing spring stiffness during walking (*p* = 0.02*, p* = 0.03). The average positive power of the exoskeleton showed no significant difference across spring stiffness conditions during running (*p* = 0.06), while the average negative power increased with increasing spring stiffness (*p* < 0.01). The detailed exoskeleton actuation parameters are presented in Additional file 1: Tables S4 and S5.

### Muscle activities

**Fig. 6:**
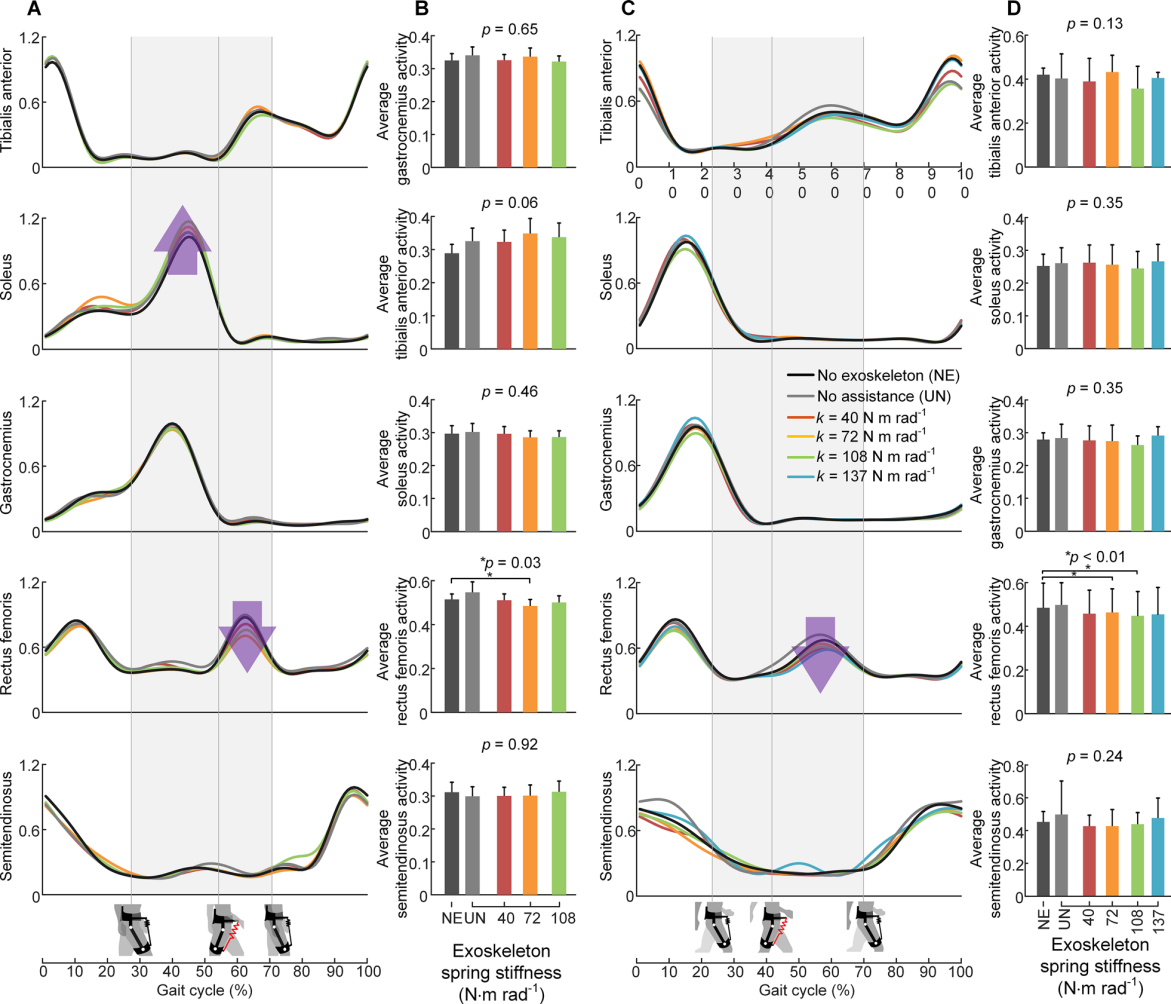
Changes in muscle activities during walking and running. **A** and **C** the activity of tibialis anterior (major ankle dorsiflexor), soleus (major ankle plantar flexor), gastrocnemius (major ankle plantar flexor), rectus femoris (major hip flexor and knee extensor) and semitendinosus (major hip extensor and knee flexor) during walking and running. **B** and **D** columns, average muscle activity over the whole gait cycle for each condition. All values were measured using electromyography and normalized to the maximum value in no exoskeleton condition (NE). *N* = 9; bars, mean; error bars, s.e.m; P values, mixed-model two-way ANOVA (random effect: participant; fixed effect: spring stiffness). *Indicates a significant difference compared to NE condition (two-sided paired t-test, *p* < 0.05)

As shown in Fig. 6A and B, the average muscle activity of the target rectus femoris showed differences across conditions (*p* = 0.03) during walking. Under the 72 N m Rad^−1^ condition, the average rectus femoris muscle activity was reduced compared to that under NE condition (two-sided paired t-test, *p* = 0.009). Under the 72 N m Rad^−1^ condition and 108 N m Rad^−1^ condition, the average muscle activity of the soleus was increased compared to NE condition (two-sided paired t-test, *p* < 0.05, *p* < 0.05). The other muscle activities were not significantly changed across conditions (mixed model two-way ANOVA, all *p* > 0.05). During running, as shown in Fig. 6C and D, the average muscle activity of the target rectus femoris was reduced under the 72 N m Rad^−1^ condition and 108 N m Rad^−1^ condition compared to that under the NE condition (two-sided paired t-test, *p* < 0.05, *p* < 0.05). The other average muscle activities were not significantly changed across conditions (mixed model two-way ANOVA, all *p* > 0.05). The detailed muscle activity parameters are presented in Additional file 1: Tables S6 and S7.

### Joint kinematics

**Fig. 7:**
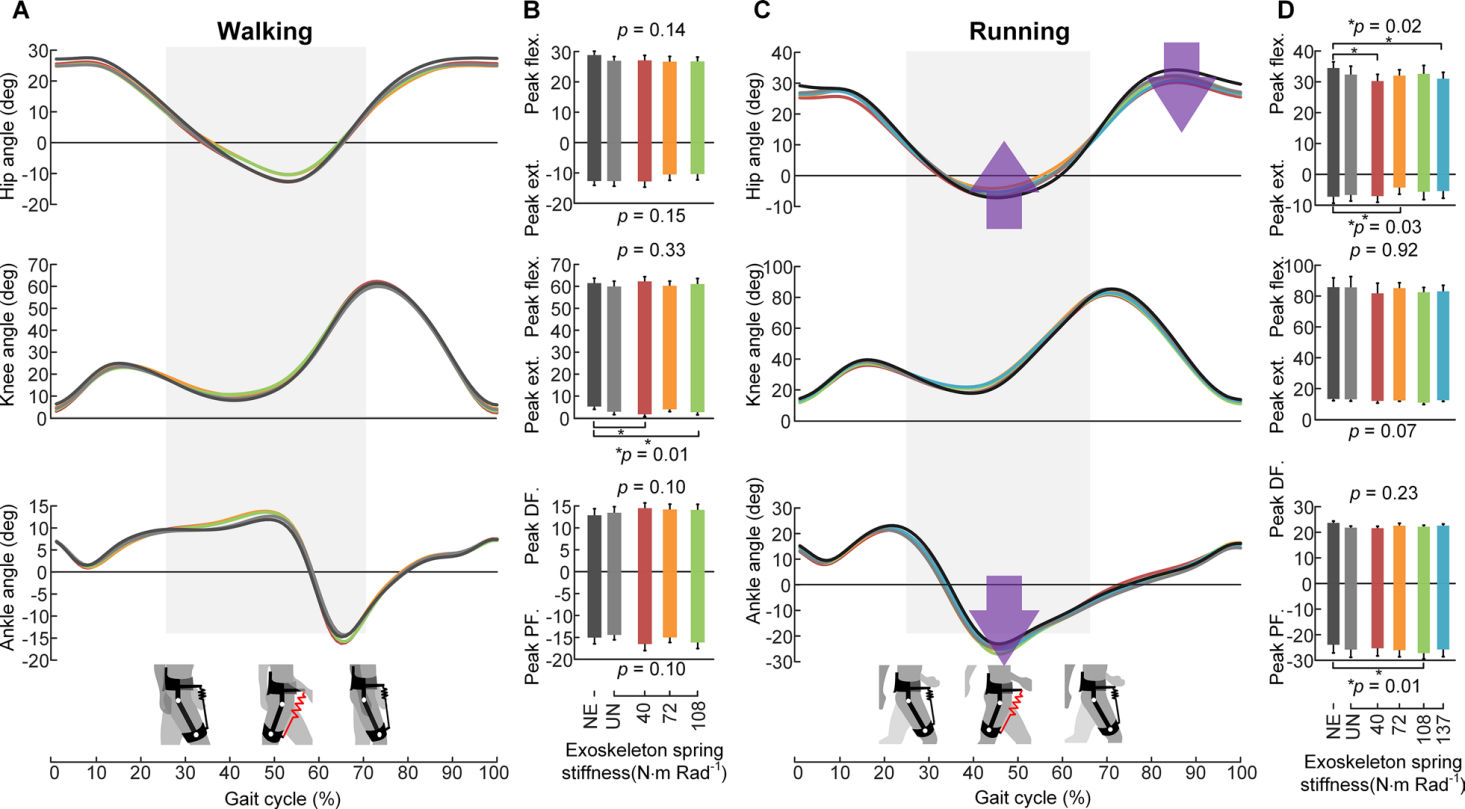
Changes in kinematics during walking and running. **A** and **C** The joint angles of hip, knee and ankle over the gait cycle, averaged by the 9 participants for each condition during walking and running. **B** and **D**, The bar graphs are the peak flexion (flex.), extension (ext.), dorsiflexion (DF.) and plantarflexion (PF.) angles for each joint and each condition. *N* = 9; bars, mean; error bars, s.e.m; *p* values, mixed-model two-way ANOVA (random effect: participant; fixed effect: spring stiffness)

As shown in Fig. 7A and B, except for the maximum knee extension angles, which showed significant differences across conditions (mixed model two-way ANOVA, *p* = 0.01), the maximum flexion/extension angles of the other joints did not show apparent changes (all *p* > 0.05) during walking. In contrast, the joint kinematics were significantly changed with the assistance of the exoskeleton during running (Fig. 7C and D). The maximum hip flexion angles (mixed model two-way ANOVA, *p* = 0.02) and extension (*p* = 0.03) angles decreased with the assistance of the exoskeleton compared to the NE condition. The maximum ankle plantarflexion angles (*p* = 0.01) increased with the assistance. The detailed joint angle parameters are presented in Additional file 1: Tables S6 and S7.

## Discussion

This study aimed to prove that it is possible to reduce the metabolic rate of both walking and running by regulating the metabolic energy of hip flexion using an unpowered hip exoskeleton. Unlike most previous exoskeletons designed based on the biomechanics of a specific gait, our exoskeleton was designed to assist hip flexion in the common energy consumption period of both walking and running. We found that the metabolic rates of walking and running can be reduced by 8.2% ± 1.5% and 9.1% ± 1.3%, corresponding to the optimal spring stiffnesses of 72 N m Rad^−1^ and 108 N m Rad^−1^ respectively. The metabolic rates of walking and running can be reduced by 7.2% ± 1.2% and 6.8% ± 1.0% with the common optimal spring stiffness. The metabolic results of walking and running at different speeds also demonstrated the generality of the proposed assistive approach. To our knowledge, this is the first unpowered exoskeleton that can be applied to both walking and running scenarios to reduce the energy expenditure at the time of submission.

The most direct reason for the metabolic reductions in both gaits is the significant decrease in the target rectus femoris (major hip flexor) activity (*p* < 0.05; *p* < 0.05). During the energy-releasing phase of the exoskeleton spring, the average muscle activity of rectus femoris showed a significant decrease compared to walking with no exoskeleton (NE), which indicated a reduction in hip flexor recruitment in both gaits with the proper spring stiffness.

The metabolic reduction showed a U-shape with increasing spring stiffness during walking and running, similar to the result of previous study on an unpowered ankle exoskeleton [7]. However, the reason for the metabolic return to the normal level might be different from theirs. In the previous study on unpowered ankle exoskeleton [7], the muscle activity of dorsiflexor counteracting exoskeleton torque was increased with assistance compared to walking without the exoskeleton. In our study, we did not find a significant increase in semitendinosus (major hip extensor) activity counteracting exoskeleton torque during walking. A possible reason why the metabolic rate of walking with 108 Nm Rad^−1^ returned to the normal level might be the increasing trend of soleus (major ankle plantar flexor) muscle activity during the energy storage phase of the exoskeleton spring (Fig. 6A). A previous biomechanical study showed that there is significant energy transfer between the ankle and hip joint during walking [31, 32]. When the exoskeleton spring passively recycles energy during hip extension, the EMG results indicated that humans might tend to increase the muscle activity of ankle plantar-flexors but not hip extensor activity to provide additional energy input for the exoskeleton. Under the optimal spring stiffness condition, the maximum metabolic reduction was likely the result of balanced muscle activity reduction in the target muscle and muscle activity increase in the soleus (major ankle plantar flexor). The excessive energy storage in high stiffness spring might result in an excessive increase in soleus activity, providing additional energy for the exoskeleton and thus offsetting the effect of assistance. Although the gastrocnemius is also a major ankle plantarflexor, its muscle activity did not show a significant change. A possible insight into this difference might be the different energetic functions between soleus and gastrocnemius. When the exoskeleton spring absorbs part of negative mechanical energy, it also passively applies hip flexion torque to the hip joint, which slows down the hip extension and the velocity of the COM. A previous simulation work showed that the energy produced by soleus was almost used to accelerate the trunk forward, whereas the energy produced by gastrocnemius was almost used to initiate leg swing [33]. During running, the human response to the proposed exoskeleton assistance was different from that during walking. The muscle activity of the soleus did not show an increasing trend as it did during walking, which might be the result of different functions of soleus between the two gaits [34]. A previous biomechanical study found a decreased contribution of the soleus to forward propulsion in running [34], which was previously found to be significant in walking [35, 36].

In our study, we tested a series of spring stiffnesses and found the optimal spring stiffnesses for walking and running. Compared to the previous parameter sweep study on an unpowered ankle exoskeleton during walking, our results showed that the optimal spring stiffness for our exoskeleton (72 N m Rad^−1^) is lower than theirs (180 N m Rad^−1^) [7]. The fact of different optimal spring stiffnesses may be related to the relatively greater quasi-stiffness of the biological ankle joint than hip joint during the assistive period. The optimal spring stiffness in our exoskeleton for walking was much greater than that of a similar unpowered exo-suit for the assistance of elderly hip flexion [37]. This outcome was possibly due to the fact that the participants in our experiments are young people within the range of 20–30 years old, which were much younger than theirs (62.1 ± 5.6 years). This phenomenon suggested that the optimal assistance magnitude of the same assistive device may vary for different age groups, especially between the young and the old, which merits further study. The optimal spring stiffness of our exoskeleton for running assistance (k = 108 Nm Rad^−1^) is also different from the optimal spring stiffness (k = 50 Nm Rad^−1^) of the unpowered hip exoskeleton proposed by Nasiri et al. [10]. Their exoskeleton exploits one leaf spring coupling to assist bilateral hip joint. The leaf spring passively provides bilateral hip joints with flexion and extension torque respectively when there is an angle difference between the bilateral hip joints. If the hip joint angle were used instead of the difference between bilateral hip joint angles, the equivalent stiffness for one leg would be approximately 100 Nm Rad^−1^. Their optimal spring stiffness might be a trade-off between the optimal spring stiffnesses for assisting both hip extension and hip flexion during the whole gait cycle, while our optimal stiffness is only for hip flexion assistance during the target assistive interval.

The metabolic results in turn demonstrated the necessity of a design method based on the analysis of the common energy consumption characteristics of the two gaits and the importance of lightweight exoskeleton structure. There have been three attempts to apply exoskeletons to assist both walking and running [10, 14, 19]. The unpowered hip exoskeleton proposed by Nasiri et al. was the first unpowered exoskeleton that can reduce the metabolic rate of running (by 8.0%) but was found to be ineffective for walking [10]. The unpowered ankle exoskeleton was the first unpowered exoskeleton that can reduce the metabolic energy (by 7.2%). When this spring-like assistance was applied to running using an emulator, the metabolic rate was increased by 11.1%. The significant differences in biomechanics are the major obstacles to these two attempts. To overcome this problem, we proposed a hip unpowered exoskeleton to regulate metabolic energy of hip flexion during the common energy consumption period of both walking and running. In our study, the metabolic rates of walking and running were reduced by 7.2% and 6.8% with the common optimal stiffness spring, overcoming the limitation of an unpowered exoskeleton being able to reduce the metabolic rate for only one gait. The best-in-class active hip exosuit, which was designed to provide customized assistance torque for both walking and running, was demonstrated to reduce the metabolic rate of walking and running by 9.3% and 4.0% respectively. Although the metabolic rate under the assistance of our exoskeleton was not reduced as much as that of their exoskeleton, the metabolic reduction of running is comparable to that of theirs. The main reason may be distributed to the lightweight exoskeleton structure. The proposed exoskeleton did not need mechanical or electronic controller, working only with hip extension and hip flexion. Moreover, the mass of the exoskeleton was concentrated close to the trunk. In this way, the metabolic penalty caused by the exoskeleton mass was minimized, especially during running.

As shown in Fig. 5, the peak exoskeleton torque did not double with the doubling of the spring stiffness. The maximum hip extension angle decreased under the stiff spring condition, which led to shorter maximum stretch length of the spring. This may be one of the reasons for the lack of doubling. A significantly changed joint angle was also found by Walsh et al. [38], supporting the viewpoint that people will alter their gait to maximize the benefit they receive. Another possible reason might be that the thigh muscle and tissue suffer more deformation under the high spring stiffness conditions. Human-device serial stiffness may be an important factor that affects the exoskeleton torque applied to humans [21]. The same trend was also found by Collins et al. [7]. In this paper, the peak exoskeleton torque did not show a significant increase between k = 310 Nm Rad^−1^ and k = 400 Nm Rad^−1^. As the muscles and tissues are softer than those of the lower leg, the musculature may suffer more deformation.

Although this study demonstrates metabolic reduction with the assistance of the proposed exoskeleton during both walking and running, we acknowledge that there are still limitations in this research. First, the precise biomechanical mechanisms underlying the metabolic reduction are still unclear, as we can only infer from the decreased muscle activity that the recruitment of the target hip flexors was reduced. In addition, the reason why exoskeleton assistance cannot reduce metabolic energy at low walking speed also remains unclear. We may gain deeper insight into the changes in the underlying muscle–tendon dynamics using in vivo ultrasound imaging techniques in future exoskeleton experiments [39].

Second, in this work, we demonstrated that the proposed assistive approach could be effective in walking and running respectively. As the results of the speed experiment demonstrated that the walking speed influenced the metabolic reduction (Fig. 4B), the optimal spring stiffness for walking assistance was also likely influenced by the walking speed. The starting time of exoskeleton assistance was approximately 10 degrees of hip flexion, consistent with the starting time of the negative power interval of the hip joint. However, the exoskeleton torque will interfere with the natural standing since the hip joint is upright in the natural standing state. We acknowledge that the exoskeleton can neither autonomously switch the assistance between the locomotion state and standing-still state nor switch the optimal assistance magnitude between walking and running as in the best-in-class powered hip exo-suit [14]. Future work may include a quasi-passive exoskeleton that uses the electronic controller to autonomously switch between the standing-still state and locomotion state.

Last, although we conducted the parameter sweep experiments to obtain the optimal exoskeleton spring stiffness that is statistically significant for metabolic reduction, the proposed exoskeleton cannot provide participants with individualized assistance, which would further reduce the metabolic energy as reported in previous studies [40, 41]. Future work may include an emulator that can actively provide spring-like torque for exploring individualized optimal assistance [19]. Collins et al. proposed an unpowered ankle exoskeleton that was demonstrated to reduce the metabolic rate of walking [7]. However, they found it ineffective to apply this passive assistance principle to assist running [19]. In contrast, they found that the metabolic rate can be reduced with high mechanical work input to the ankle using an emulator. Besides, Walsh et al. found that the metabolic rate of running can be reduced with the hip extension assistance of a powered hip exosuit [14]. Combined with these findings, the metabolic rate of running may be further reduced with net mechanical work input to assist hip flexion.

## Conclusions

This paper first demonstrated that the metabolic cost of walking and running could be reduced by an unpowered hip exoskeleton with a universal assistive principle of assisting hip flexion. The design method, based on analyzing the common energy consumption characteristics between the two gaits and kinesiology of the human musculoskeletal system, may inspire future unpowered or powered exoskeleton design for the assistance of diverse daily gaits. The results of different changes in muscle activities provided a new insight into the human response to the same assistive principle for different gaits (both walking and running).

## Supplementary Information

**Additional file 1: Figure S1.** Musculoskeletal structure of ankle plantar-flexors and hip flexors. **Figure S2.** Exoskeleton structure parameters. **Table S1.** Net metabolic rates of walking and running on the treadmill. **Table S2.** Net metabolic rates of walking and running at different speeds. **Table S3.** Net metabolic rates of walking and running with common optimal stiffness spring. **Table S4.** Exoskeleton actuation parameters during walking. **Table S5.** Exoskeleton actuation parameters during running. **Table S6.** Biomechanical parameters during walking. **Table S7.** Biomechanical parameters during running.

## Data Availability

All data generated or analyzed in this study are included in this published article and Additional file 1.

## Abbreviations

ANOVA: Analyses of variance
NE: No exoskeleton condition
UN: Walking/running with exoskeleton but unassisted condition
COM: Center of mass
s.t.d: Standard deviation of mean
s.e.m: Standard error of mean
