# Supplementary material for: Reducing the metabolic energy of walking and running using an unpowered hip exoskeleton
Source: J Neuroeng Rehabil. 2021 Jun 6;18:95. doi: 10.1186/s12984-021-00893-5 (PMC8182901; doi:10.1186/s12984-021-00893-5)
Supplement: Supplementary file 1 — Additional file 1: Figure S1. Musculoskeletal structure of ankle plantar-flexors and hip flexors. Figure S2. Exoskeleton structure parameters. Table S1. Net metabolic rates of walking and running on the treadmill. Table S2. Net metabolic rates of walking and running at different speeds. Table S3. Net metabolic rates of walking and running with common optimal stiffness spring. Table S4. Exoskeleton actuation parameters during walking. Table S5. Exoskeleton actuation parameters during running. Table S6. Biomechanical parameters during walking. Table S7. Biomechanical parameters during running. [file 12984_2021_893_MOESM1_ESM.docx]

**Additional material**

**The PDF file includes:**

Fig. S1

Fig. S2

Table S1-S7

**Additional**


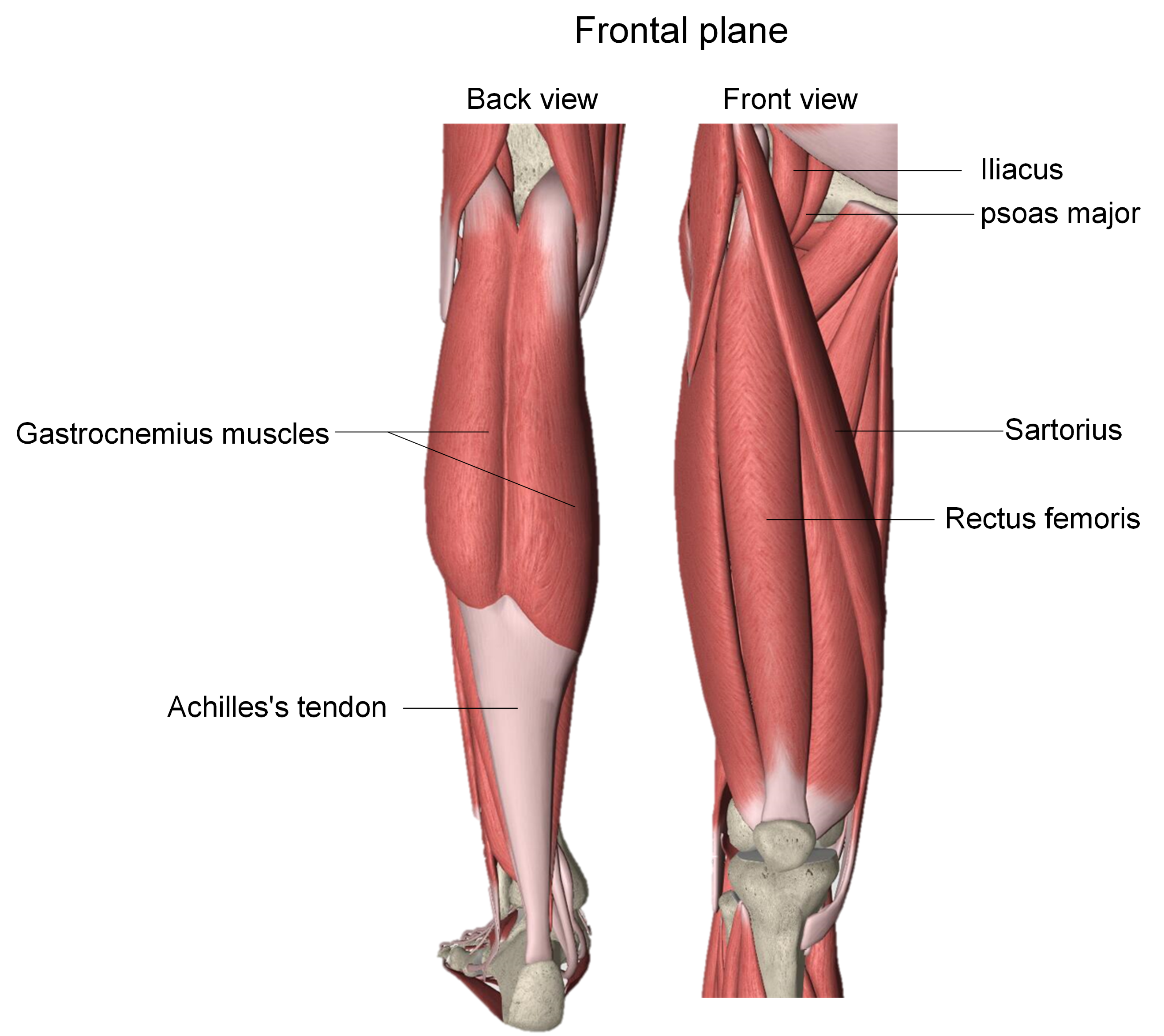


**Fig. S1. Musculoskeletal structure of ankle plantarflexors and hip flexors.** Gastrocnemius muscles (left), which is the main ankle plantarflexor muscle group, acts in series with Achilles’s tendon. The Achilles’s tendon is the most elastic and energy efficient component in human body. Compared to gastrocnemius muscles, most hip flexors (right) do not work in series with such a long elastic tendon.

**Calculation process of exoskeleton torque**

As shown in Fig. S2A and Fig. S2B, we set the length of lever component, connecting rod, thigh rod and thigh brace as L1 = 0.27m, L2 = 0.14m, L3 = 0.3m, L4 = 0.09m. The lever component is perpendicular to the connecting rod. The thigh brace is perpendicular to thigh rod. We can calculate the real lever length (D_lever_) through trigonometric functions as follows:


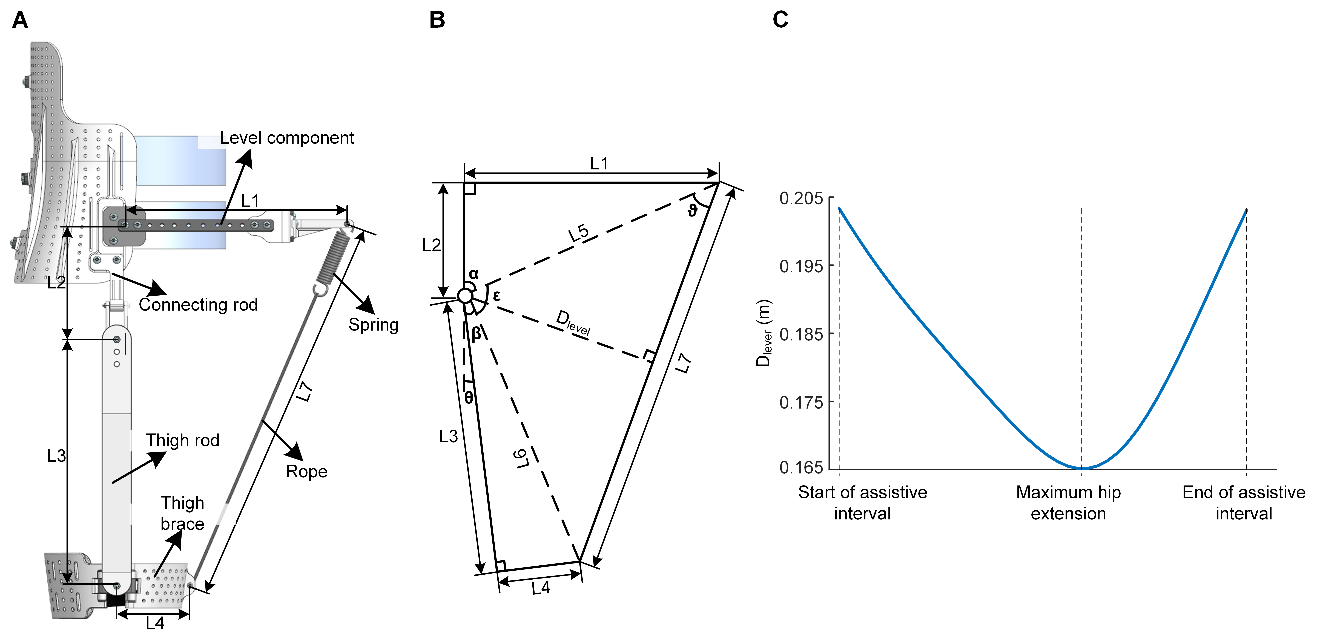


**Fig. S2 Exoskeleton structure parameters. (A)** Exoskeleton components. **(B)** Structural dimensions of each exoskeleton part. **(C)** The length of lever arm in the assistive interval of one participant as an example.

$\text{L5}\text{ }\text{=}\text{ }\sqrt{\text{L}\text{1}^{\text{2}}\text{+L}\text{2}^{\text{2}}}$ (1)

$\text{L6}\text{ }\text{=}\text{ }\sqrt{\text{L}\text{3}^{\text{2}}\text{+L}\text{4}^{\text{2}}}$ (2)

$\text{α}\text{ }\text{=}\text{ }\arctan\text{L1/L2}$ (3)

$\text{β}\text{ }\text{=}\text{ }\arctan\text{L4/L3}$ (4)

$\text{ε}\text{ }\text{= π- α-β-θ}$ (5)

Then we can obtain L7 through (1)-(6) functions:

$\text{L7}\text{ }\text{= }\sqrt{\text{L}\text{5}^{\text{2}}\text{+L}\text{6}^{\text{2}}\text{-2*L5*L6*cos(ε)}}$ (6)

Then we can obtain D_lever_ through (7)-(8) functions:

$\frac{\text{L6}}{\text{sinϑ}}\text{ }\text{= }\frac{\text{L7}}{\text{sinε}}$ (7)

$\text{D}_{\text{leve}\text{r}}\text{ }\text{=}\text{ }\text{L5*}\sin\left( \text{ϑ} \right)\text{=L5*L6*sin(ϵ)/L7}$ (8)

After obtaining the D_lever_, we obtain the exoskeleton torque ($\text{τ}_{\text{exo}}$) by multiplying load cell force by D_lever_

$$\text{τ}_{\text{exo}}\text{ }\text{=}\text{ }\text{force*}\text{D}_{\text{leve}\text{r}}$$

As shown in Fig. S2C, the D_lever_ varies with the hip angle (θ), we recalculated the average equivalent rotational spring stiffness using the average length of the lever arm in the assistance interval. We first calculated the mean length of the force lever for each participant and each stiffness condition. Then, the data were averaged across conditions and across participants. The average length of force lever is $\bar{D}$_lever_ = 0.19m. The average equivalent rotational spring stiffnesses are 40, 72, 108, 137 Nm Rad^-1^.

**Table S1. Net metabolic rates of walking and running on the treadmill.**

We used two-sided paired t-test with Holm-Šidák correction to compare spring conditions to NE (no exoskeleton) condition to find which spring condition had a significant reduction in metabolic rate (*N* = 9, significant level α = 0.05, * indicated statistically difference between two conditions).

| **Participant** | **Net metabolic rate (W kg^-1^)** | | | | | | | | | | |
| --- | --- | --- | --- | --- | --- | --- | --- | --- | --- | --- | --- |
|  | **Walking at 1.5 m s^-1^** | | | | | **Running at 2.5 m s^-1^** | | | | | |
|  | **NE** | **UN** | **40** | **72** | **108** | **NE** | **UN** | **40** | **72** | **108** | **137** |
| 1 | 3.978 | 4.525 | 3.809 | 3.678 | 4.540 | 8.473 | 8.536 | 8.395 | 8.088 | 7.756 | 8.001 |
| 2 | 3.790 | 3.736 | 3.432 | 3.194 | 3.780 | 8.801 | 8.916` | 8.512 | 8.434 | 8.284 | 8.647 |
| 3 | 3.490 | 3.398 | 3.736 | 3.286 | 3.403 | 8.773 | 8.988 | 8.391 | 7.928 | 7.795 | 8.591 |
| 4 | 4.554 | 4.540 | 4.245 | 4.003 | 4.124 | 8.166 | 8.811 | 9.143 | 8.032 | 7.034 | 8.559 |
| 5 | 3.194 | 3.194 | 2.904 | 2.889 | 3.122 | 10.466 | 11.147 | 9.817 | 9.555 | 9.012 | 10.232 |
| 6 | 2.986 | 2.914 | 2.928 | 2.928 | 3.049 | 10.372 | 11.087 | 10.049 | 10.071 | 10.005 | 10.293 |
| 7 | 4.613 | 4.680 | 4.487 | 4.400 | 4.859 | 9.838 | 10.119 | 9.645 | 9.532 | 9.173 | 9.649 |
| 8 | 3.528 | 3.722 | 3.393 | 3.369 | 3.480 | 9.473 | 9.692 | 9.409 | 9.186 | 8.878 | 9.745 |
| 9 | 3.378 | 3.315 | 3.219 | 2.962 | 3.272 | 10.374 | 10.464 | 10.553 | 9.739 | 9.105 | 10.316 |
| **Mean** $\text{±}$ **SEM** | **3.723**$\text{±}$ **0.190** | **3.780**$\text{±}$ **0.217** | **3.573**$\text{±}$ **0.183*** | **3.412**$\text{±}$ **0.174*** | **3.73**$\text{±}$ **0.214** | **9.415**$\text{±}$  **0.297** | **9.751**$\text{±}$  **0.334** | **9.324**$\text{±}$  **0.259** | **8.952**$\text{±}$  **0.277*** | **8.560**$\text{±}$  **0.305*** | **9.337**$\text{±}$  **0.297** |

**Table S2. Net metabolic rates of walking and running at different speeds.**

We used two-sided paired t-test to compare optimal spring condition (EXO: 72Nm Rad^-1^ for walking; 108 Nm Rad^-1^ for running) to no exoskeleton (NE) condition for each walking/running speed to identify whether EXO condition had a significant reduction in metabolic rate (*N* = 9, significant level α = 0.05, * indicated statistically difference from NE condition).

| **Participant** | **Net metabolic rate (W kg^-1^)** | | | | | | | | | | | | | |
| --- | --- | --- | --- | --- | --- | --- | --- | --- | --- | --- | --- | --- | --- | --- |
|  | **Walking speed (m s^-1^)** | | | | | | | | **Running speed (m s^-1^)** | | | | | |
|  | **1.0** | | **1.25** | | **1.5** | | **1.75** | | **2.0** | | **2.25** | | **2.5** | |
|  | **EXO** | **NE** | **EXO** | **NE** | **EXO** | **NE** | **EXO** | **NE** | **EXO** | **NE** | **EXO** | **NE** | **EXO** | **NE** |
| 1 | 1.597 | 1.617 | 3.151 | 3.267 | 3.94 | 4.511 | 5.45 | 6.002 | 8.731 | 9.244 | 10.401 | 11.451 | 11.684 | 12.923 |
| 2 | 1.225 | 1.22 | 2.265 | 2.367 | 2.952 | 3.209 | 4.264 | 4.603 | 7.12 | 8.518 | 8.523 | 8.78 | 9.133 | 9.515 |
| 3 | 1.888 | 1.917 | 2.962 | 3.069 | 3.703 | 3.896 | 5.116 | 5.392 | 8.199 | 8.993 | 9.762 | 10.75 | 10.967 | 11.906 |
| 4 | 2.246 | 2.251 | 2.633 | 2.73 | 3.291 | 3.625 | 4.554 | 5.014 | 7.299 | 8.533 | 8.693 | 9.907 | 9.762 | 11.234 |
| 5 | 1.205 | 1.152 | 2.222 | 2.226 | 2.938 | 3.035 | 4.825 | 5.276 | 8.615 | 9.293 | 10.246 | 10.963 | 10.551 | 11.408 |
| 6 | 2.125 | 2.168 | 2.401 | 2.643 | 3.006 | 3.306 | 4.158 | 4.724 | 6.66 | 7.11 | 7.933 | 8.969 | 8.91 | 10.338 |
| 7 | 1.486 | 1.486 | 2.289 | 2.493 | 3.015 | 3.577 | 4.637 | 5.426 | 7.681 | 8.155 | 7.928 | 9.365 | 10.314 | 11.635 |
| 8 | 1.592 | 1.646 | 1.96 | 2.013 | 2.592 | 2.667 | 4.004 | 4.259 | 6.827 | 7.362 | 6.873 | 7.163 | 8.955 | 9.864 |
| 9 | 1.423 | 1.384 | 2.017 | 2.163 | 2.665 | 2.875 | 4.114 | 4.53 | 7.012 | 7.521 | 7.059 | 7.817 | 9.196 | 9.796 |
| **Mean** $\text{±}$ **SEM** | **1.643**$\text{±}$ **0.124** | **1.649**$\text{±}$  **0.131** | **2.433**$\text{±}$  **0.136*** | **2.552**$\text{±}$  **0.140** | **3.122**$\text{±}$  **0.15*** | **3.411**$\text{±}$  **0.188** | **4.569**$\text{±}$  **0.164*** | **5.025**$\text{±}$  **0.183** | **7.572**$\text{±}$  **0.259*** | **8.303**$\text{±}$  **0.273** | **8.602**$\text{±}$  **0.434*** | **9.463**$\text{±}$  **0.483** | **9.941**$\text{±}$  **0.331*** | **10.958**$\text{±}$  **0.382** |

**Table S3. Net metabolic rates of walking and running with common optimal stiffness spring.**

We used two-sided paired t-test to compare optimal spring condition (83 Nm Rad^-1^) to no exoskeleton (NE) condition to identify whether EXO condition had a significant reduction in metabolic rate (*N* = 9, significant level α = 0.05, * indicated statistically difference between two conditions).

|  | **Net metabolic rate (W kg^-1^)** | | | |
| --- | --- | --- | --- | --- |
| **Participants** | **Walking (1.5m s^-1^)** | | **Running (2.5m s^-1^)** | |
|  | 83 Nm Rad^-1^ | NE | 83 Nm Rad^-1^ | NE |
| 1 | 4.012 | 4.525 | 12.095 | 12.913 |
| 2 | 3.175 | 3.427 | 9.370 | 9.554 |
| 3 | 3.611 | 3.698 | 10.900 | 11.601 |
| 4 | 3.33 | 3.606 | 9.859 | 11.127 |
| 5 | 3.248 | 3.475 | 10.619 | 11.234 |
| 6 | 2.957 | 3.088 | 9.636 | 10.135 |
| 7 | 3.156 | 3.514 | 10.454 | 11.62 |
| 8 | 2.783 | 2.977 | 9.06 | 9.762 |
| 9 | 2.831 | 3.059 | 8.654 | 9.341 |
| **Mean** $\text{±}$ **SEM** | 3.324 ± 0.130* | 3.485 ± 0.156 | 10.072 ± 0.353* | 10.810 ± 0.396 |

**Table S4. Exoskeleton actuation parameters during walking.** Mean $\text{±}$ standard errors of actuation timing and mechanics parameters for 3 spring stiffnesses conditions during walking. #, * and † and indicate statistical significance for the 40 N🞌m Rad^-1^ vs. 72 N🞌m Rad^-1^, 40 N🞌m Rad^-1^ vs. 108 N🞌m Rad^-1^ and 72 N🞌m Rad^-1^ vs. 108 N🞌m Rad^-1^ comparisons

|  | 40 N🞌m Rad^-1^ | 72 N🞌m Rad^-1^ | 108 N🞌m Rad^-1^ |  |
| --- | --- | --- | --- | --- |
| Assistance start timing (% GC) | 28.3 ± 1.9 | 28.6 ± 2.0 | 27.1 ± 1.8 |  |
| Assistance end timing (% GC) | 69.6 ± 1.0 | 69.7 ± 1.1 | 70.7 ± 0.9 |  |
| Peak moment timing (% GC) | 56.3 ± 0.7 | 56.3 ± 0.6 | 56.0 ± 0.6 |  |
| Exoskeleton peak moment (N🞌m kg^-1^) | 0.083 ± 0.011 | 0.099 ± 0.011 | 0.121 ± 0.012 | #*† |
| Exoskeleton average moment (N🞌m kg^-1^) | 0.021 ± 0.004 | 0.026 ± 0.004 | 0.030 ± 0.004 | #*† |
| Exoskeleton peak positive power (W kg^-1^) | 0.171 ± 0.023 | 0.183 ± 0.027 | 0.226 ± 0.027 | *† |
| Exoskeleton peak negative power (W kg^-1^) | -0.080 ± 0.015 | -0.089 ± 0.017 | -0.108 ± 0.018 | *† |
| Average positive work rate (W kg^-1^) | 0.018 ± 0.003 | 0.019 ± 0.003 | 0.023 ± 0.004 | *† |
| Average negative work rate (W kg^-1^) | -0.013 ± 0.003 | -0.013 ± 0.003 | -0.018 ± 0.003 | *† |

**Table S5. Exoskeleton assistance parameters during running.** Mean $\text{±}$ standard errors of actuation timing and mechanics parameters for 4 spring stiffnesses conditions during running. #, *, † , ‡, ¥ and ¢ indicate statistical significance for the 40 N🞌m Rad^-1^ vs. 72 N🞌m Rad^-1^, 40 N🞌m Rad^-1^ vs. 108 N🞌m Rad^-1^, 40 N🞌m Rad^-1^ vs. 137 N🞌m Rad^-1^, 72 N🞌m Rad^-1^ vs. 108 N🞌m Rad^-1^, 72 N🞌m Rad^-1^ vs. 137 N🞌m Rad^-1^ and 108 N🞌m Rad^-1^ vs. 137 N🞌m Rad^-1^ comparisons.

|  | 40 N🞌m Rad^-1^ | 72 N🞌m Rad^-1^ | 108 N🞌m Rad^-1^ | 137 N🞌m Rad^-1^ |  |
| --- | --- | --- | --- | --- | --- |
| Assistance start timing (%GC) | 21.7 ± 0.6 | 21.8 ± 0.4 | 21.4 ± 0.4 | 20.7 ± 0.5 |  |
| Assistance end timing (%GC) | 66.7 ± 1.2 | 67.8 ± 0.9 | 68.3 ± 1.0 | 69.0 ± 0.8 |  |
| Peak moment timing (%GC) | 41.2 ± 0.5 | 41.7 ± 0.9 | 40.3 ± 0.9 | 40.6 ± 0.9 |  |
| Exoskeleton peak moment (N🞌m kg^-1^) | 0.077 ± 0.007 | 0.099 ± 0.006 | 0.111 ± 0.007 | 0.123 ± 0.006 | *†‡¥¢ |
| Exoskeleton average moment (N🞌m kg^-1^) | 0.022 ± 0.003 | 0.026 ± 0.002 | 0.030 ± 0.002 | 0.034 ± 0.002 | #*†‡¥¢ |
| Exoskeleton peak positive power (W kg^-1^) | 0.086 ± 0.011 | 0.103 ± 0.01 | 0.100 ± 0.010 | 0.118 ± 0.007 |  |
| Exoskeleton peak negative power (W kg^-1^) | -0.144 ± 0.018 | -0.159 ± 0.02 | -0.196 ± 0.019 | -0.213 ± 0.022 | *†¥¢ |
| Average positive work rate (W kg^-1^) | 0.015 ± 0.003 | 0.017 ± 0.002 | 0.017 ± 0.002 | 0.021 ± 0.002 |  |
| Average negative work rate (W kg^-1^) | -0.019 ± 0.003 | -0.020 ± 0.003 | -0.026 ± 0.003 | -0.030 ± 0.003 | †¥ |

**Table S6. Biomechanical parameters during walking.** Mean $\text{±}$ standard errors of spatio-temporal parameters, muscle activity parameters and kinematical parameters for no exoskeleton condition (NE), no assistance condition (UN) and 3 stiffness conditions. * indicated statistically difference from NE (two-sided paired t-test, *N* = 9, significant level α = 0.05).

|  | **NE** | **UN** | **40 Nm Rad^-1^** | **72 Nm Rad^-1^** | **137 Nm Rad^-1^** |
| --- | --- | --- | --- | --- | --- |
| **Stride length (m)** | 1.43 ± 0.02 | 1.43 ± 0.02 | 1.45 ± 0.02 | 1.44 ± 0.02 | 1.43 ± 0.02 |
| **Average muscle activity** |  |  |  |  |  |
| Soleus | 0.289 ± 0.027 | 0.325 ± 0.039 | 0.337 ± 0.043 | 0.349 ± 0.045 | 0.323 ± 0.036 |
| Gastrocnemius | 0.297 ± 0.024 | 0.302 ± 0.026 | 0.287 ± 0.019 | 0.286 ± 0.02 | 0.297 ± 0.022 |
| Tibialis anterior | 0.325 ± 0.021 | 0.34 ± 0.026 | 0.322 ± 0.017 | 0.337 ± 0.026 | 0.326 ± 0.017 |
| Rectus femoris | 0.515 ± 0.025 | 0.547 ± 0.047 | 0.501 ± 0.03 | 0.485 ± 0.029* | 0.511 ± 0.029 |
| Semitendinosus | 0.312 ± 0.03 | 0.299 ± 0.029 | 0.313 ± 0.032 | 0.301 ± 0.032 | 0.301 ± 0.026 |
| **Peak joint angle (°)** |  |  |  |  |  |
| Hip extension | -12.68 ± 1.40 | -12.69 ± 1.67 | -12.80 ± 1.87 | -10.49 ± 1.93 | -10.37 ± 1.91 |
| Hip flexion | 28.82 ± 1.28 | 26.97 ± 1.36 | 27.1 0 ± 1.61 | 26.65 ± 1.74 | 26.76 ± 1.42 |
| Knee extension | 5.21 ± 1.22 | 2.87 ± 1.32 | 1.92 ± 1.29* | 3.97 ± 1.05 | 2.66 ± 1.20* |
| Knee flexion | 61.45 ± 2.25 | 59.95 ± 2.40 | 62.28 ± 2.10 | 60.25 ± 2.11 | 61.07 ± 2.51 |
| Ankle dorsiflexion | 12.88 ± 1.48 | 13.45 ± 1.39 | 14.49 ± 1.20 | 14.21 ± 1.21 | 14.12 ± 1.26 |
| Ankle plantarflexion | -15.05 ± 1.41 | -14.44 ± 1.11 | -16.52 ± 1.49 | -15.00 ± 1.14 | -16.15 ± 1.39 |

**Table S7: Biomechanical parameters during running.** Mean $\text{±}$ standard errors of spatio-temporal parameters, muscle activity parameters and kinematical parameters for no exoskeleton condition (NE), no assistance condition (UN) and 3 stiffness conditions. * indicated statistically difference from NE condition (two-sided paired t-test, *N* = 9, significant level α = 0.05).

|  | **NE** | **UN** | **40 N m Rad^-1^** | **72 N m Rad^-1^** | **108 N m Rad^-1^** | **137 N m Rad^-1^** |
| --- | --- | --- | --- | --- | --- | --- |
| **Stride length (m)** | 1.79 ± 0.04 | 1.73 ± 0.05* | 1.8 ± 0.04 | 1.77 ± 0.03 | 1.81 ± 0.04 | 1.76 ± 0.03 |
| **Averaged muscle activity** |  |  |  |  |  |  |
| Soleus | 0.420 ± 0.010 | 0.403 ± 0.037 | 0.389 ± 0.035 | 0.432 ± 0.025 | 0.405 ± 0.009 | 0.357 ± 0.034 |
| Gastrocnemius | 0.252 ± 0.012 | 0.261 ± 0.016 | 0.262 ± 0.018 | 0.256 ± 0.020 | 0.266 ± 0.017 | 0.245 ± 0.017 |
| Tibialis anterior | 0.279 ± 0.007 | 0.284 ± 0.014 | 0.277 ± 0.015 | 0.274 ± 0.016 | 0.291 ± 0.009 | 0.262 ± 0.009 |
| Rectus femoris | 0.485 ± 0.038 | 0.498 ± 0.034 | 0.458 ± 0.036 | 0.463 ± 0.036* | 0.449 ± 0.037* | 0.454 ± 0.041 |
| Semitendinosus | 0.453 ± 0.021 | 0.498 ± 0.068 | 0.426 ± 0.022 | 0.427 ± 0.034 | 0.439 ± 0024 | 0.477 ± 0.041 |
| **Peak joint angle (°)** |  |  |  |  |  |  |
| Hip extension | -7.25 ± 2.11 | -6.68 ± 1.98 | -7.06 ± 1.98 | -4.28 ± 2.14* | -5.64 ± 2.55 | -5.41 ± 2.33 |
| Hip flexion | 34.47 ± 2.02 | 32.35 ± 2.72 | 30.25 ± 2.17* | 32.01 ± 1.89 | 32.6 ± 2.74 | 31.03 ± 2.05* |
| Knee extension | 13.25 ± 1.08 | 13.05 ± 1.18 | 11.97 ± 1.33 | 12.28 ± 0.47 | 10.93 ± 1.22 | 12.42 ± 0.71 |
| Knee flexion | 85.77 ± 6.00 | 85.65 ± 6.98 | 81.77 ± 6.57 | 85.17 ± 3.47 | 82.51 ± 3.09 | 83.06 ± 3.91 |
| Ankle dorsiflexion | 23.67 ± 0.70 | 21.81 ± 0.58 | 21.59 ± 0.74 | 22.59 ± 0.91 | 22.18 ± 0.58 | 22.58 ± 0.67 |
| Ankle plantarflexion | -23.90 ± 3.20 | -25.82 ± 3.03 | -25.27 ± 3.00 | -26.05 ± 2.58 | -27.17 ± 2.28* | -25.77 ± 2.79 |
